# Supplementary material for: Functional optic tract rewiring via subtype- and target-specific axonal regeneration and presynaptic activity enhancement
Source: Nat Commun. 2025 Mar 4;16:2174. doi: 10.1038/s41467-025-57445-x (PMC11880380; doi:10.1038/s41467-025-57445-x)
Supplement: Supplementary file 1 — Supplementary Information [file 41467_2025_57445_MOESM1_ESM.pdf]

Supplementary Fig. 1

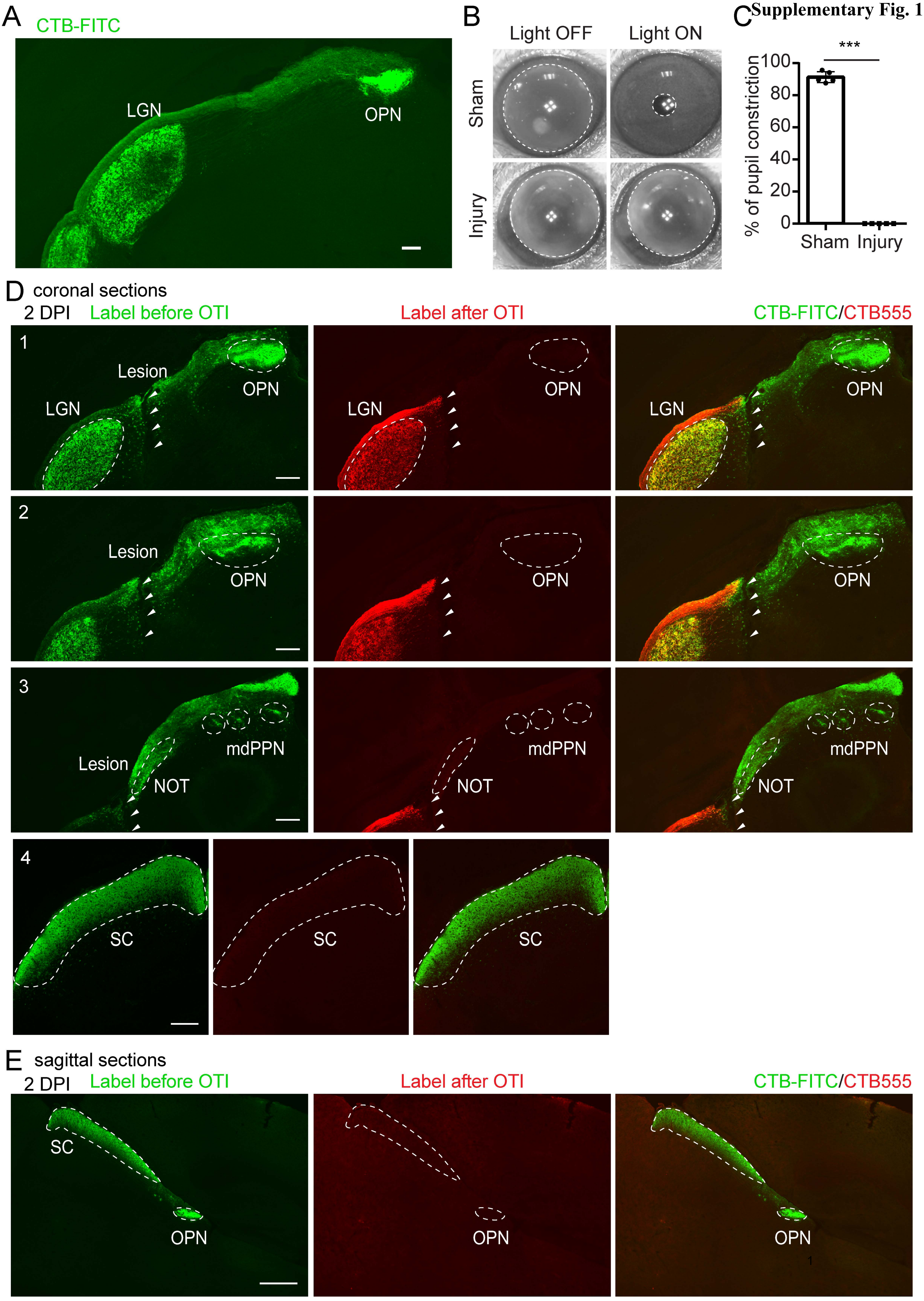

Supplementary Fig. 1: Verification of the complete pre-OPN OTI.

(A) Fluorescence images showing the labeling of the LGN and OPN regions through intravitreal injection of CTB-FITC (green) in intact mice. Scale bar: 200 µm.

(B) Representative pupil images from sham and one-day pre-OPN OTI mice before and after 1-minute light (0.5 mW/cm^2) stimulation. The upper panel shows normal PLR (91%) in the sham mice, whereas the pre-OPN injured mice show no response.

(C) Quantification of PLR constriction following 1-minute light stimulation. Data presents mean ± SEM. n=5 mice. Statistical significance: $p<0.0001$, Student's t-test, two-sided.

(D-E) Fluorescence images of coronal (D) and sagittal (E) sections showing post-OTI CTB-555 (red) injection did not label RGC axons projecting beyond the lesion site, which was pre-labeled with CTB-FITC (green). All axons projecting beyond the LGN, including those targeting the OPN, NOT, mdPPN, and SC, were crushed. The arrowhead indicates the lesion site. Scale bar: 200 µm (D), 500 µm (E).

*** $p\leq0.001$

Source data are provided as a Source Data file.

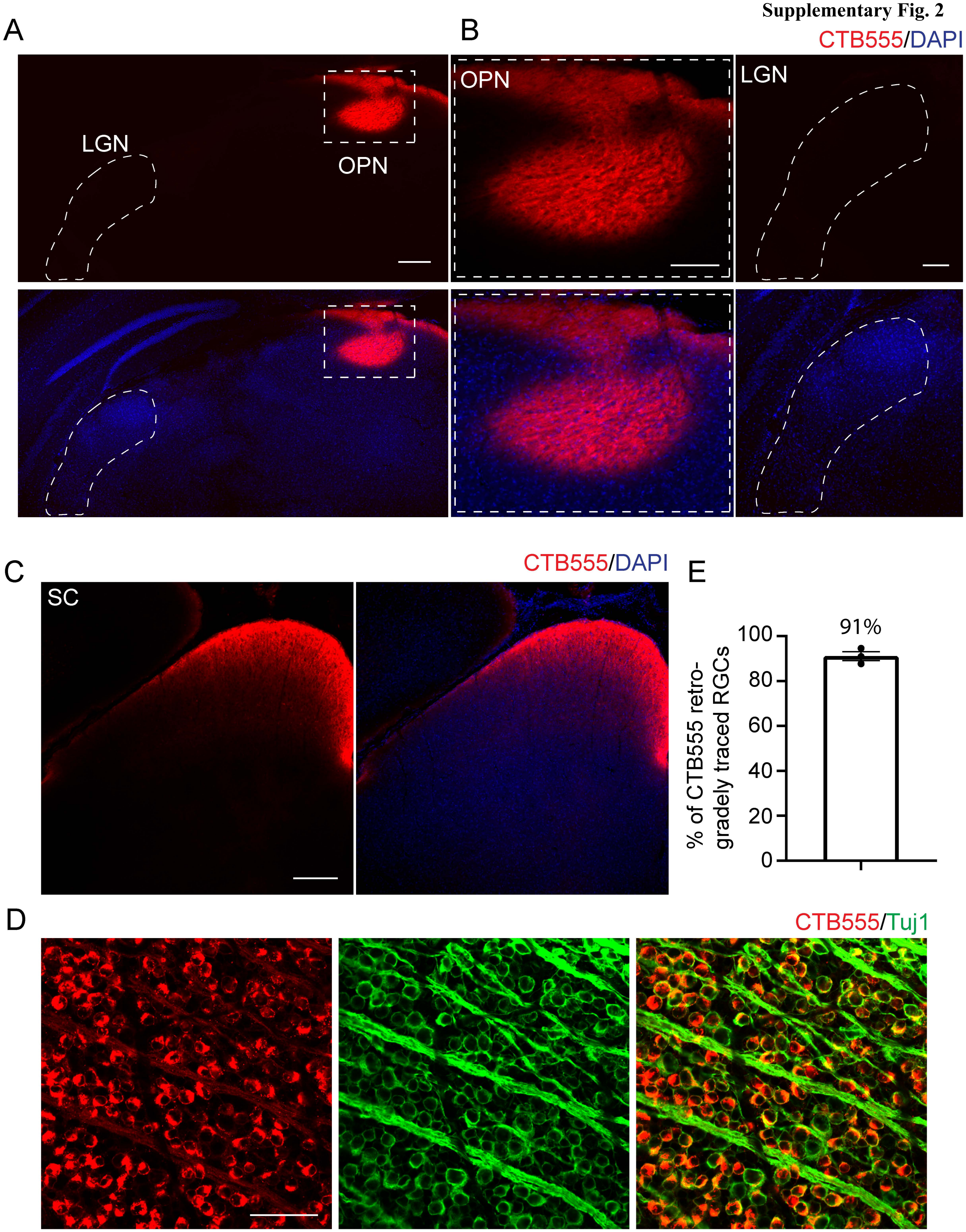
A
B
CTB555/DAPI
LGN
OPN
OPN
LGN
C
CTB555/DAPI
SC
E
% of CTB555 retro-gradely traced RGCs
91%
100
80
60
40
20
0
D
CTB555/Tuj1

Supplementary Fig. 2: Retrograde labeling of RGCs that project beyond LGN.

(A-C) Representative images showing the injection sites of the retrograde tracer CTB555 (red) (A, OPN; B, enlarged OPN; C, SC). Scale bar: (A) 200 μm, (B) 100 μm, (C) 200 μm.

(D) Representative images of the flat-mounted retina showing the retrogradely labeled RGCs by CTB555 (red) co-localized with RGC marker Tuj1 (green). Scale bar: 50 μm.

(E) Quantification of the percentage of the retrogradely labeled RGCs that project beyond LGN for (D). Data presents mean ± SEM. n=3 mice.

Source data are provided as a Source Data file.

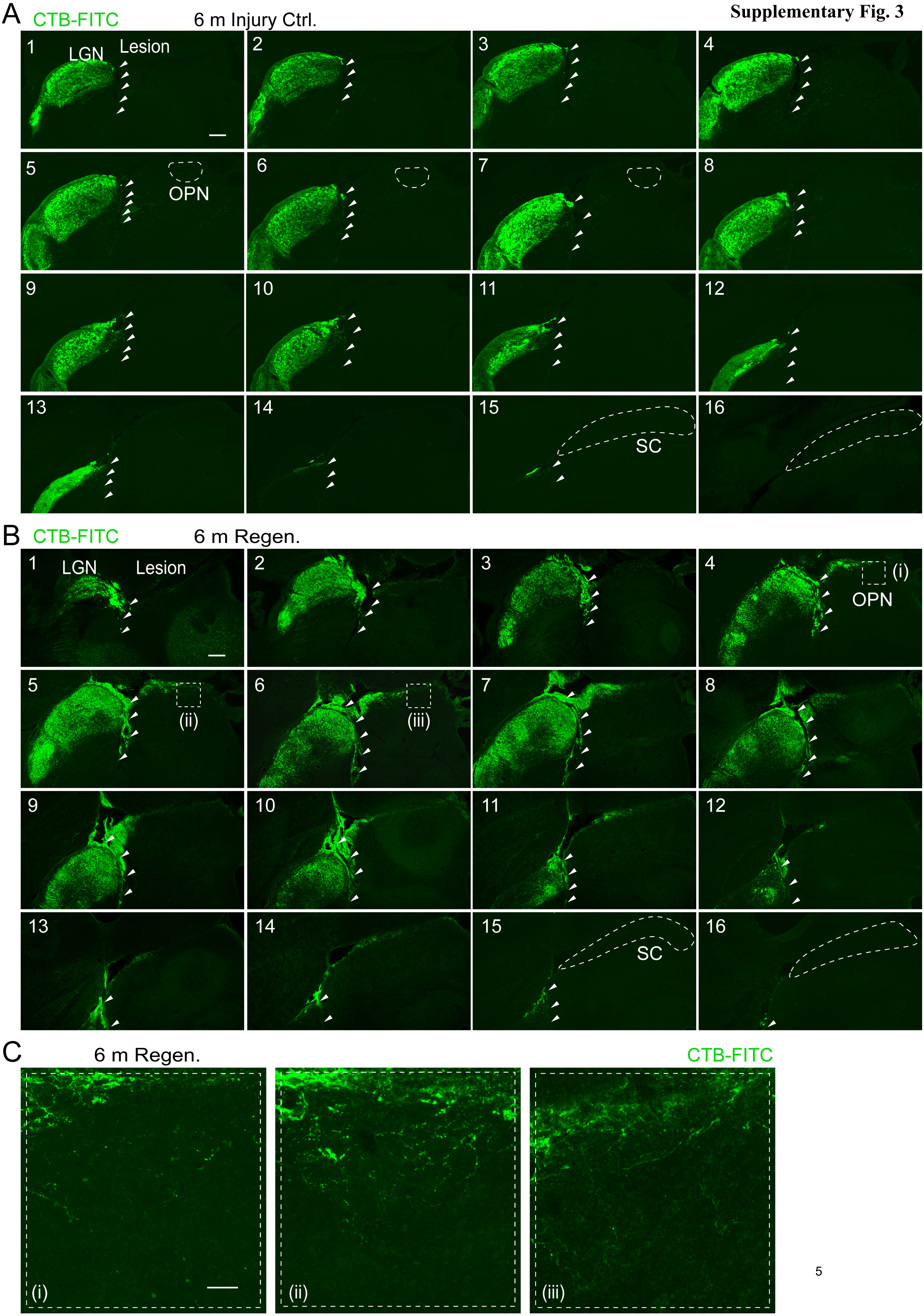
A
CTB-FITC
6 m Injury Ctrl.
1
LGN
Lesion
2
3
4
5
OPN
6
7
8
9
10
11
12
13
14
15
SC
16
B
CTB-FITC
6 m Regen.
1
LGN
Lesion
2
3
4
(i)
OPN
5
(ii)
6
(iii)
7
8
9
10
11
12
13
14
15
SC
16
C
6 m Regen.
CTB-FITC
(i)
(ii)
(iii)

Supplementary Fig. 3: Serial coronal sections in 6m Injury Ctrl. and Regen. mice.

(A) Serial sections of anterior to posterior coronal images from 6m Injury Ctrl. mice injected with AAV2-LacZ showed no axons beyond the lesion site after 6 months of injury. The arrowhead indicates the lesion site. The dash lines outline the OPN regions (5-7) and SC regions (15-16) in Injury Ctrl mouse. Scale bar: 200 μm.

(B) Serial sections of anterior to posterior coronal images from Pten/Socs3 knockout mice displayed regenerated axons passing across the lesion site and growing into OPN after 6 months of injury. The OPN regions (4-6) and SC regions (15-16) in 6 m Regen. mouse are outlined by dash lines. Scale bar: 200 μm.

(C) Zoomed-in images of rectangles-indicated areas in (B) showing regenerated RGC axons in the OPN regions (i-iii, B4-6). Scale bar: 50 μm.

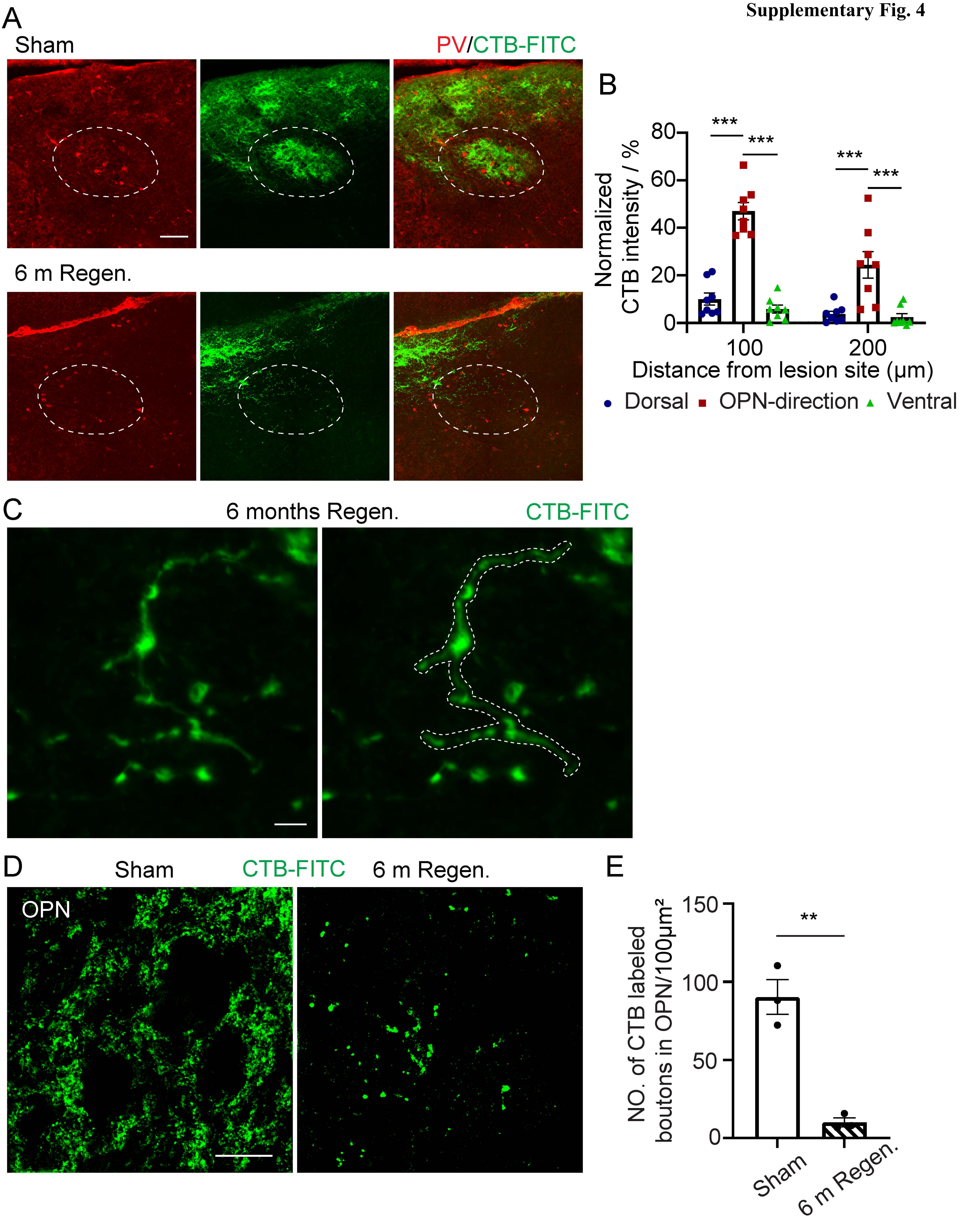
A
Sham
PV/CTB-FITC
6 m Regen.
B
Normalized CTB intensity / %
80
60
40
20
0
***
***
***
***
100
200
Distance from lesion site (μm)
Dorsal
OPN-direction
Ventral
C
6 months Regen.
CTB-FITC
D
Sham
CTB-FITC
6 m Regen.
OPN
E
NO. of CTB labeled boutons in OPN/100μm²
150
100
50
0
**
Sham
6 m Regen.

Supplementary Fig. 4: Parvalbumin staining to delineate the OPN region and the characteristics of the regenerated axons.

(A) Representative images of parvalbumin (PV) staining (red) in brain sections illustrating the OPN from Sham and 6 m Regen. group. The shown brain regions were contralateral to the CTB-FITC (green) injected eye. Scale bar: 100 μm.

(B) Quantification of the percentage of regenerating axons that grew dorsally, ventrally, or along the original trajectory toward the OPN was conducted using normalized CTB intensity in animals from the 6 m Regen. group. Data presents mean ± SEM. n=8 mice. Statistical significance: 100 μm: $p<0.0001$ (Dosal vs. OPN-direction, and OPN-direction vs. Ventral), 200 μm: $p<0.0001$ (Dosal vs. OPN-direction, and OPN-direction vs. Ventral), ANOVA followed by Bonferroni test, two-sided.

(C) One example of CTB-labeled regenerated axons (green) beyond the lesion site includes collaterals. The right panel outlines the regenerated axons with a dashed line. Scale bar: 5 μm.

(D) Representative images show the CTB-labeled axon terminal boutons (green) within the OPN from Sham and 6 m Regen. mice. Scale bar: 10 μm.

(E) Quantification of the density of CTB-labeled axon terminal boutons in the OPN for both Sham and 6 m Regen. mice. Data presents mean ± SEM. n=3 mice. Statistical significance: $p=0.0021$, Student's t-test, two-sided.

** $p\leq0.01$, *** $p\leq0.001$

Source data are provided as a Source Data file.

Supplementary Fig. 5

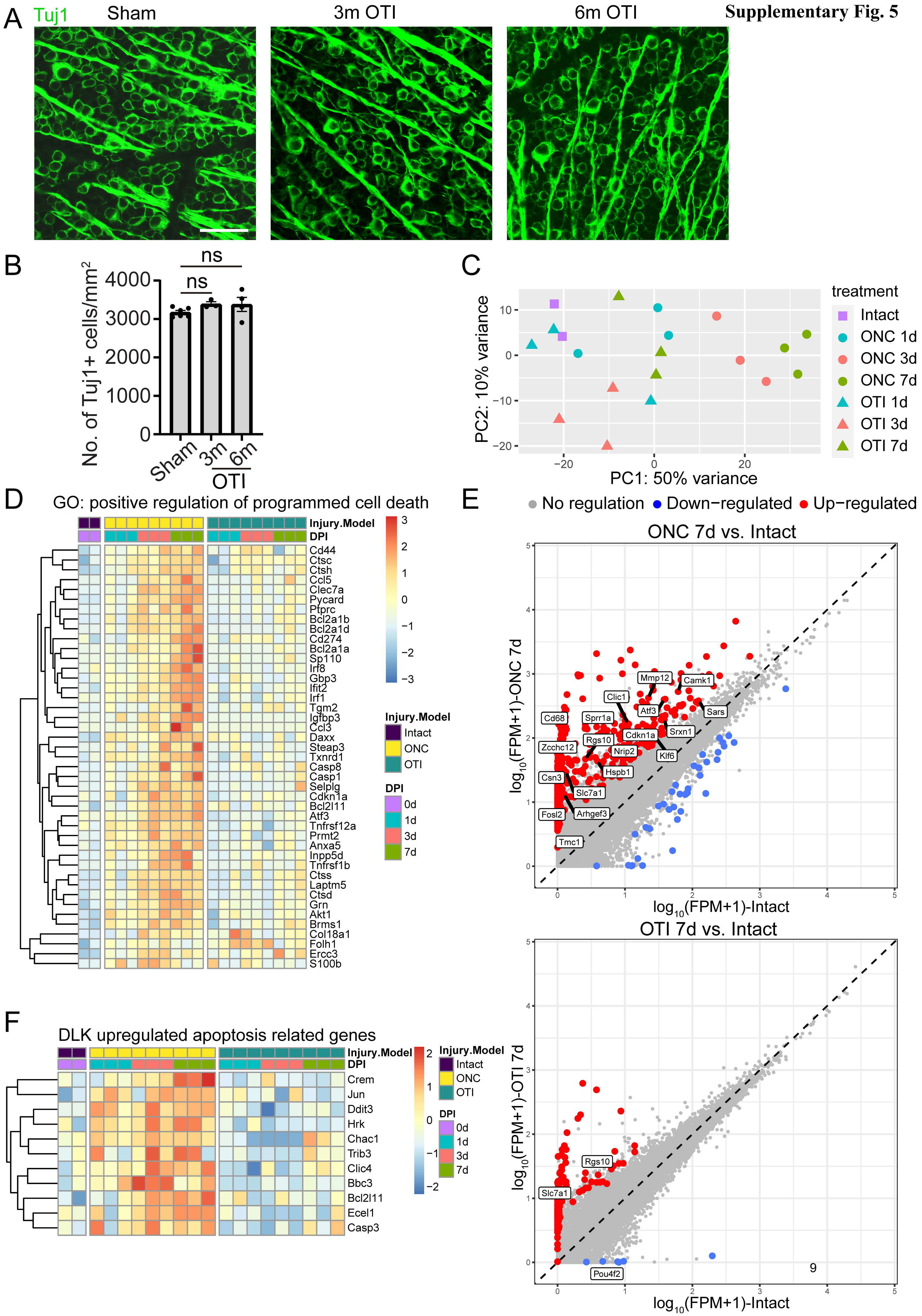

Supplementary Fig. 5: RGC survived the pre-OPN OTI and identification of cell death-related pathways via bulk RNA sequencing.

(A) Representative images of Tuj1 staining (green) in whole-mount retinas from sham mice, as well as from 3 months and 6 months pre-OPN OTI mice. Scale bar: 50 μm.

(B) Quantification of the number of surviving RGCs. Data presents mean ± SEM. n=6 (Sham), n=3 (3 m OTI), and n=4 (6 m OTI). Statistical significance: p=0.3345 (sham vs. 3 m OTI), 0.3084 (sham vs. 6 m OTI), ANOVA followed by Dunnett test, two-sided.

(C) PCA plot of bulk RNAseq experiment showing the separation of optic tract injury (OTI) and optic nerve crush (ONC) groups.

(D) Heatmap showing the expression of genes from "GO: positive regulation of programmed cell death" between the OTI model and ONC model from the bulk RNAseq data.

(E) Scatterplot showing the differences in terms of differentially expressed genes (red/blue dot labeled) and specifically DLK-dependent genes (text labeled) between different injury models.

(F) Heatmap showing the expression of DLK upregulated apoptosis-related genes between the OTI model and ONC model from the bulk RNAseq data. These genes show obvious activation in the ONC model but not in the OTI model.

ns, not significant p>0.05

Source data are provided as a Source Data file.

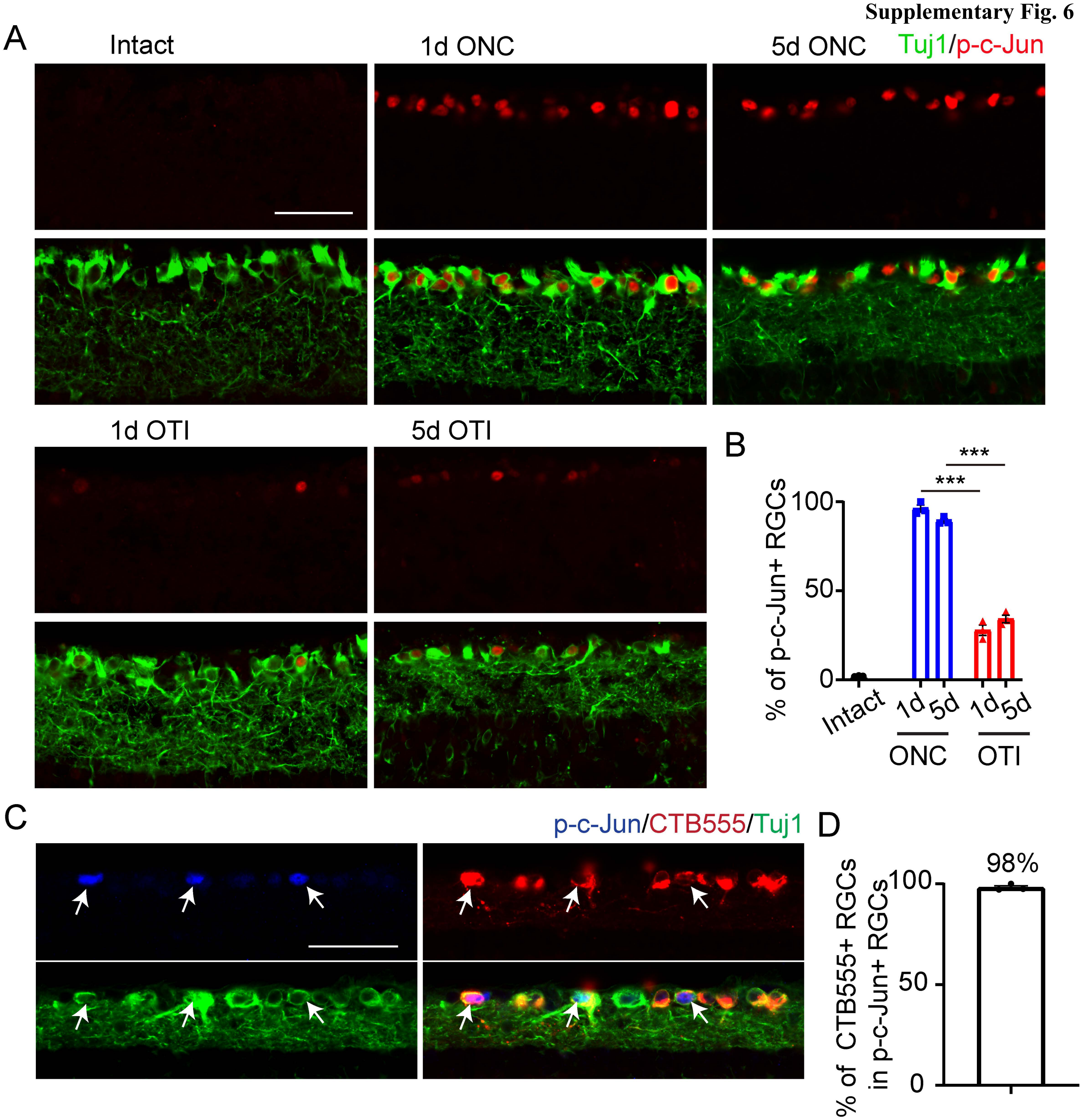
A
Intact
1d ONC
5d ONC
Tuj1/p-c-Jun
1d OTI
5d OTI
B
% of p-c-Jun+ RGCs
100
50
0
***
***
Intact
1d
5d
1d
5d
ONC
OTI
C
p-c-Jun/CTB555/Tuj1
D
% of CTB555+ RGCs in p-c-Jun+ RGCs
98%
100
50
0

Supplementary Fig. 6: OTI induces lower DLK activation and p-c-Jun expression is upregulated in RGCs that were injured by the OTI.

(A) Representative images of Tuj1(green) and p-c-Jun (red) double staining in sectioned retinas to show the DLK-pathway activation status at different times after ONC and OTI. Scale bar: 50 μm.

(B) Quantification of the percentage of p-c-Jun+ RGCs across intact, ONC, and OTI groups. Data presents mean ± SEM. n=3 mice. Statistical significance: $p<0.0001$, ANOVA followed by Tukey's test, two-sided.

(C) Representative images illustrate the colocalization of p-c-Jun (blue) and OTI-affected RGCs labeled by CTB555 (red) in mice at 5 days post-OTI. Tuj1 staining (green) highlights all RGCs. White arrows indicate OTI-activated p-c-Jun+ RGCs that co-localize with CTB555+ RGCs. Scale bar: 50 μm.

(D) Quantification of the percentage of CTB555+ RGCs among p-c-Jun+ RGCs. Data are presented as mean ± SEM. n = 3 mice.

*** $p\leq 0.001$

Source data are provided as a Source Data file.

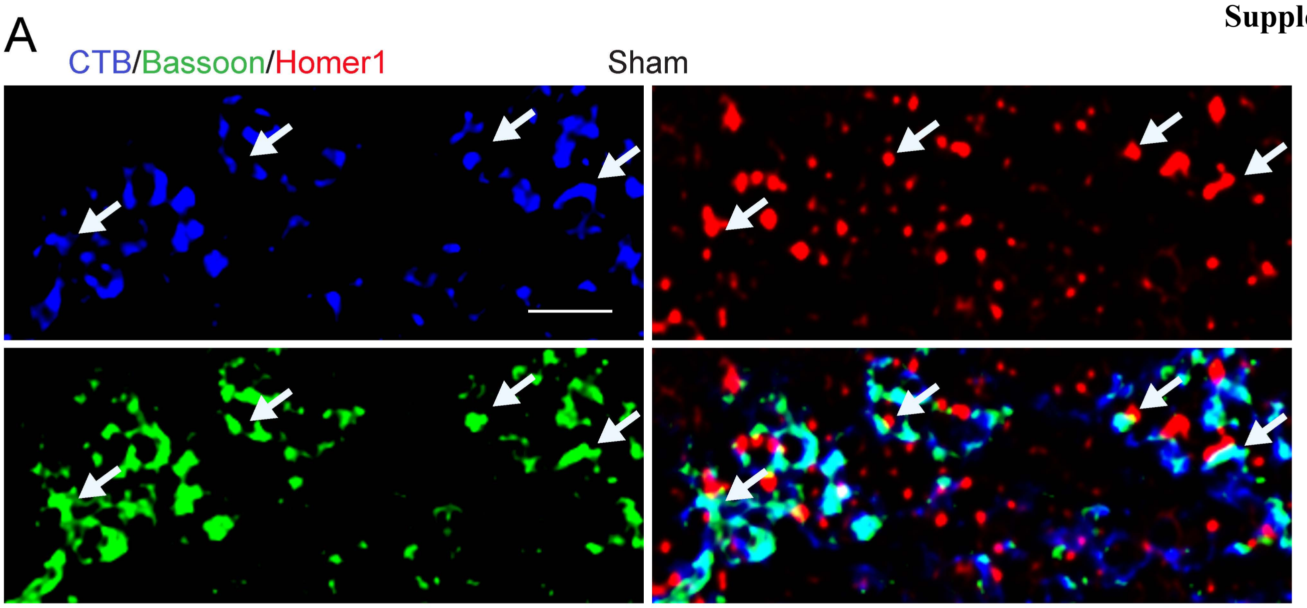
A
CTB/Bassoon/Homer1
Sham

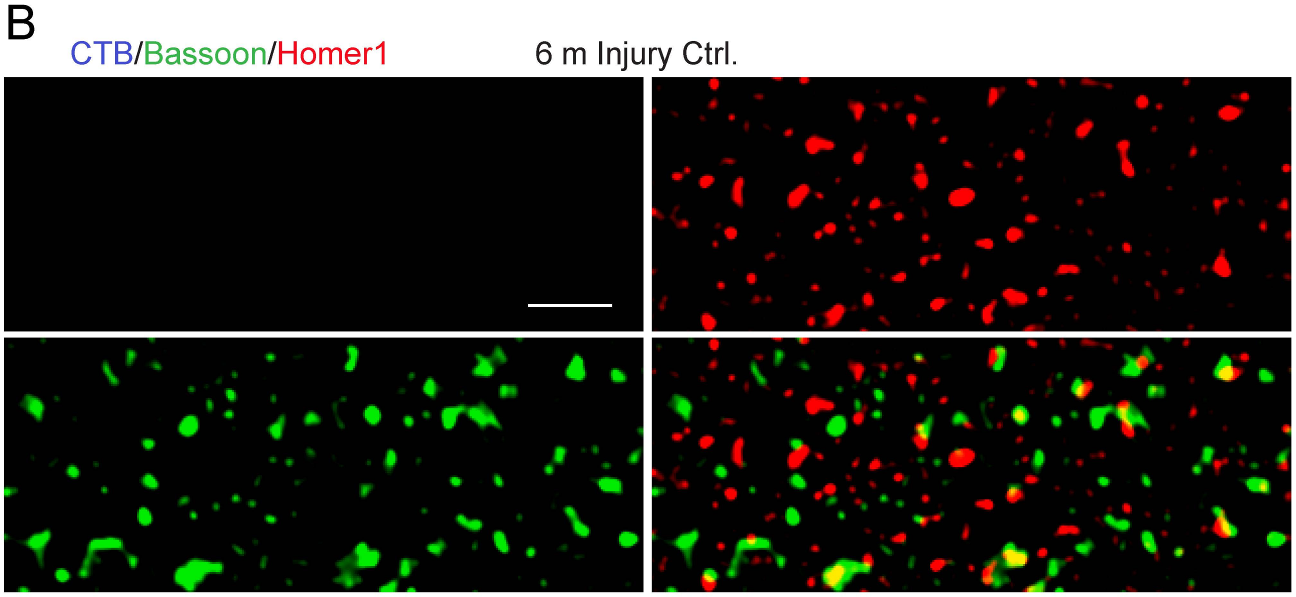
B
CTB/Bassoon/Homer1
6 m Injury Ctrl.

Supplementary Fig. 7: Examination of anatomical synapses in OPN from the Sham and 6 m Injury Ctrl. mice.

(A) Representative images showing CTB-labeled axon terminals (blue) were colocalized with Bassoon (green) and adjacent to Homer1 (red) in the OPN of Sham mice. Arrows pinpoint synapses in OPN. Scale bar: 2 μm.

(B) Representative images showing the absence of CTB-labeled synapses in the OPN region of Injury Ctrl. mice 6 months after pre-OPN OTI. Scale bar: 2 μm.

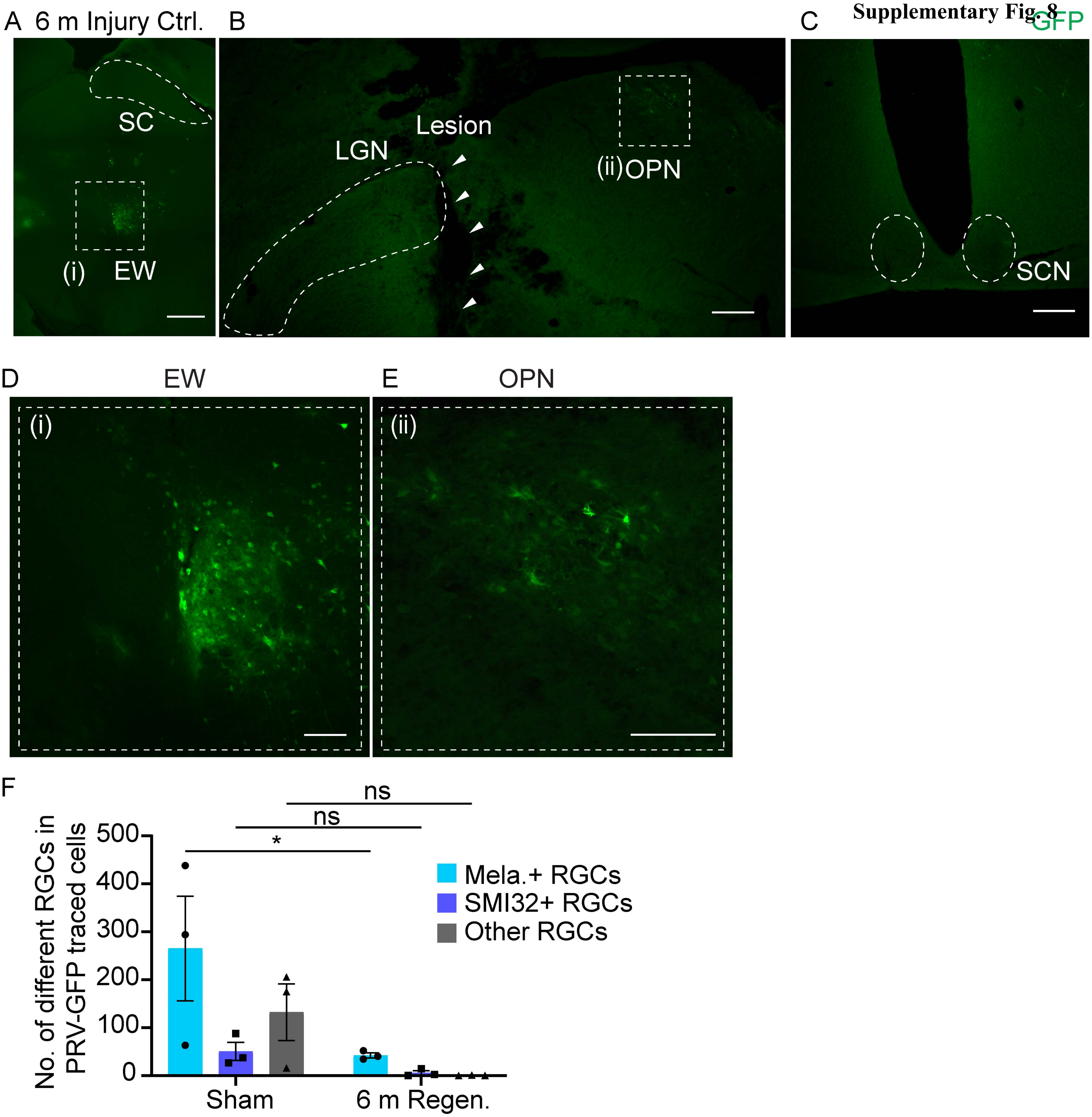
A 6 m Injury Ctrl.
SC
(i) EW
B
Lesion
LGN
(ii)OPN
C
GFP
SCN
D EW
(i)
E OPN
(ii)
F
No. of different RGCs in PRV-GFP traced cells
500
400
300
200
100
0
Sham
6 m Regen.
ns
ns
*
Mela.+ RGCs
SMI32+ RGCs
Other RGCs

Supplementary Fig. 8: PRV-labeled brain regions in 6 m Injury Ctrl. mice and the number of different types of RGC in PRV-GFP traced cells.

(A-C) Representative images showing PRV-GFP retrogradely traced brain nuclei in the major RGC projection regions of the Injury Ctrl. mice, outlined with dashed lines. These regions include the SC (A), EW (A), OPN (B), LGN (B), and SCN (C). White arrowheads indicate the lesion site in (B). Scale bars: (A) 500 μm, (B) 200 μm, (C) 200 μm.

(D-E) Representative zoomed-in images showing PRV-GFP-labeled cells in the EW (i, D) and OPN (ii, E). Scale bars: (D) 100 μm, (E) 100 μm.

(F) Quantification of the number of PRV-GFP-traced melanopsin+ ipRGCs, SMI32+ αRGCs, and other RGC types. Data presents mean ± SEM. n=3 mice. Statistical significance: p=0.029 (Mela. + RGCs), p>0.999 (SMI32+ RGCs), p=0.2834 (Other RGCs). ANOVA followed by Bonferroni test, two-sided.

ns, not significant p>0.05, * p≤0.05

Source data are provided as a Source Data file.

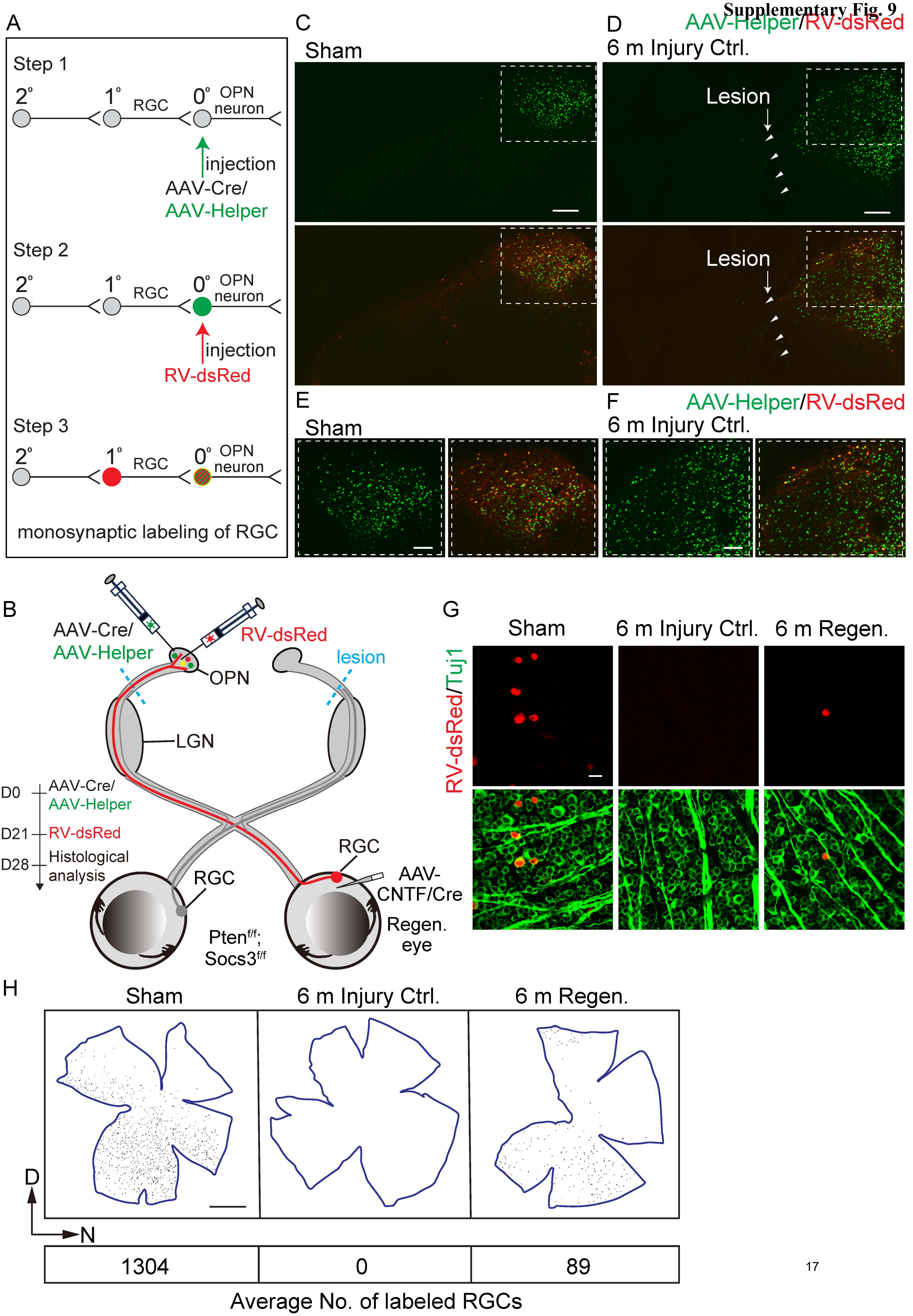
A
Step 1
2°
1°
RGC
0°
OPN neuron
injection
AAV-Cre/
AAV-Helper
Step 2
2°
1°
RGC
0°
OPN neuron
injection
RV-dsRed
Step 3
2°
1°
RGC
0°
OPN neuron
monosynaptic labeling of RGC
C
Sham
D
AAV-Helper/RV-dsRed
6 m Injury Ctrl.
Lesion
Lesion
E
Sham
F
AAV-Helper/RV-dsRed
6 m Injury Ctrl.
B
AAV-Cre/
AAV-Helper
RV-dsRed
lesion
OPN
LGN
D0
AAV-Cre/
AAV-Helper
D21
RV-dsRed
D28
Histological analysis
RGC
RGC
AAV-CNTF/Cre
Regen. eye
Pten f/f; Socs3 f/f
G
Sham
6 m Injury Ctrl.
6 m Regen.
RV-dsRed/Tuj1
H
Sham
6 m Injury Ctrl.
6 m Regen.
D
N
1304
0
89
Average No. of labeled RGCs

Supplementary Fig. 9: Monosynaptic rabies virus tracing of synapses reestablishing RGCs.

(A) A diagram illustrating the steps and labeling outcomes of retrograde monosynaptic tracing using the rabies virus. The injection of AAV-Cre and AAV-Helper (AAV-DIO-EGFP-TVA, AAV-DIO-RVG) results in the labeling of the 0^0 OPN neurons with GFP. Following this, the injection of RV-dsRed labels both the 0^0 OPN neurons and the 1^0 RGCs, which forms a monosynaptic connection with the 0^0 OPN neurons, with dsRed, while the 2^0 neurons remain unlabeled.

(B) A schematic representation illustrates rabies virus retrograde tracing from the OPN to the cell bodies of RGCs in the eye injected with AAV-CNTF/Cre. The rabies virus identifies RGCs whose regenerated axons successfully extended to the OPN and reformed synaptic connections.

(C-D) Fluorescence images depict the injection site in Sham (C) and 6 m Injury Ctrl. (D) mice. The helper viruses and AAV1-Syn-Cre were injected into the OPN of the mice (green). Three weeks after the helper virus injection, RV-EnvA-ΔG-dsRed (red) was injected into the OPN. The arrowhead indicates the lesion site in (D). Scale bar: 200 µm.

(E-F) Zoomed-in images of the areas indicated by rectangles in (C) and (D), showing the injection site in the OPN region. Scale bar: 100 µm.

(G) Fluorescence images display RV-dsRed-traced cells (red) co-localized with the RGC marker Tuj1 (green) in Sham and 6 m Regen. mice. Scale bar: 20 µm.

(H) Flat-mount retina images show RV-traced RGCs (black dots) across different groups. Scale bar: 1000 µm. The lower panel presents the average number of traced RGCs in each group. n=6 (Sham) or 3 (6 m Injury Ctrl. and 6 m Regen.) mice per group.

Source data are provided as a Source Data file.

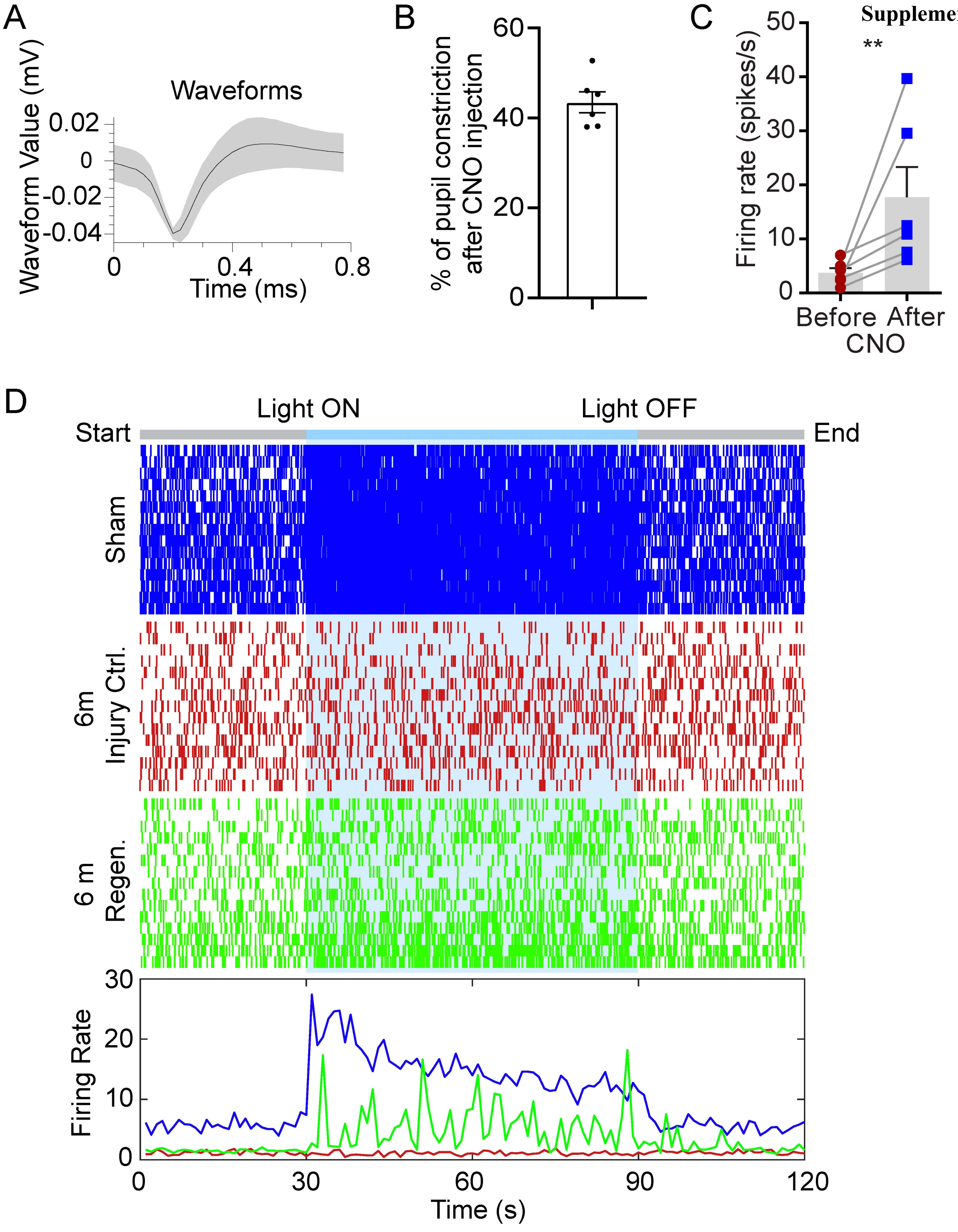
A
Waveforms
Waveform Value (mV)
0.02
0
-0.02
-0.04
0
0.4
0.8
Time (ms)
B
% of pupil constriction after CNO injection
60
40
20
0
C
**
Firing rate (spikes/s)
50
40
30
20
10
0
Before
After
CNO
D
Light ON
Light OFF
Start
End
Sham
6m Injury Ctrl.
6 m Regen.
Firing Rate
30
20
10
0
0
30
60
90
120
Time (s)

Supplementary Fig. 10: Neural activity dynamics of the OPN neurons across different groups.

(A) Schematic image showing the spike shape recorded in the OPN.

(B) Quantification of pupil constriction after 5 mg/kg CNO intraperitoneal injection. The mice received AAV-hSyn-DREADD-Gq injection into the OPN. The PLR test was performed in the dark. Data presents mean ± SEM. n=6 mice.

(C) Quantification of the neuronal firing rate in the OPN before and after 5 mg/kg CNO treatment. The mice received AAV-hSyn-DREADD-Gq injection into the OPN. Data presents mean ± SEM. n=6 mice. Statistical significance: $p=0.0046$, Ratio paired t-test, two-sided.

(D) In-vivo electrophysiology recording of OPN neural signals and kinematics. The upper three panels show the raster plots of typical OPN neurons in response to 15 times of 60 s light-dark stimulation from Sham mice, 6 m Injury Ctrl. mice and 6 m Regen. mice. The light blue rectangle indicates the 60 s duration of light stimulation. The fourth panel shows the average firing rate across 15 trials among different groups.

** $p \leq 0.01$

Source data are provided as a Source Data file.

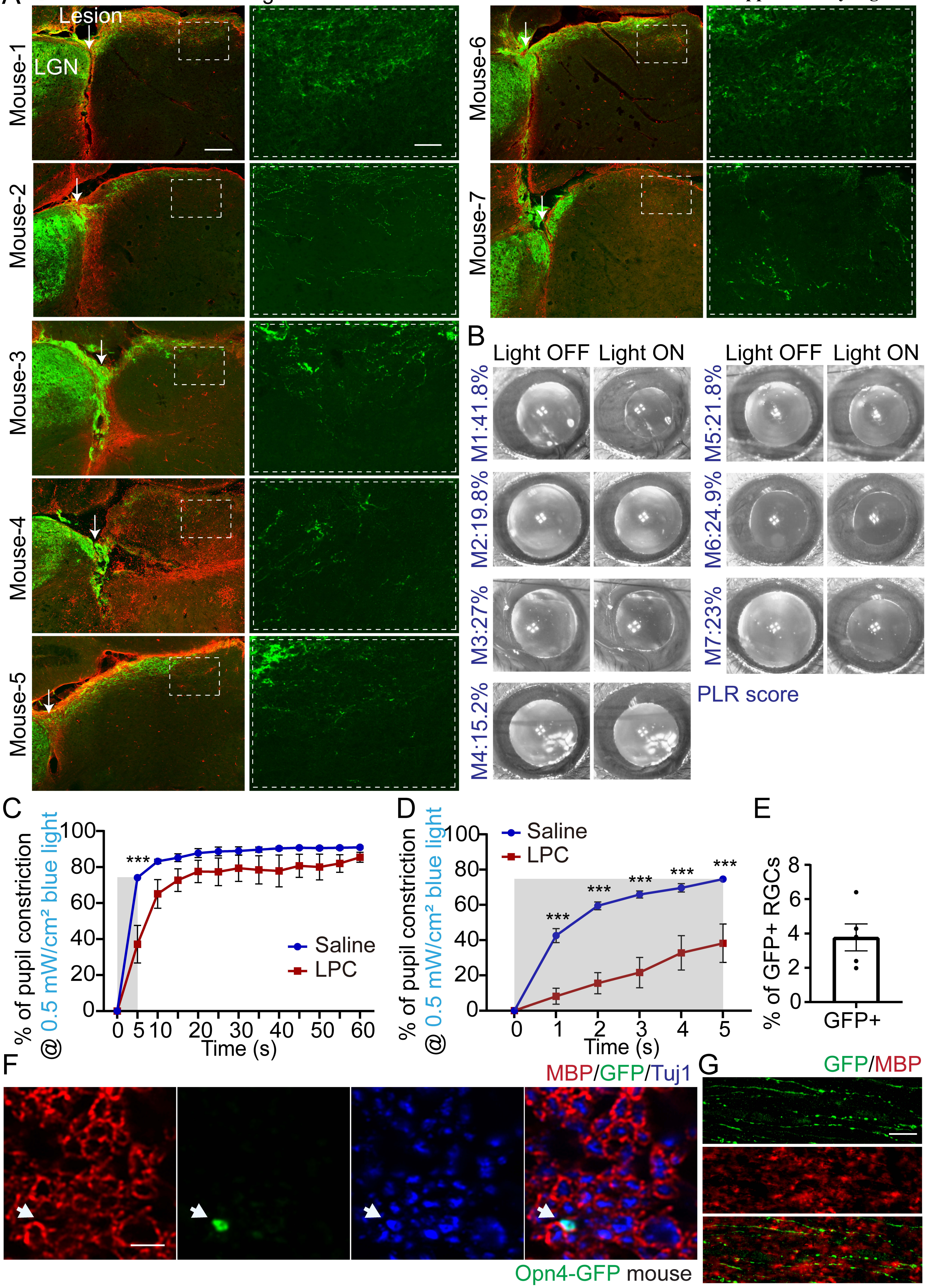
A
CTB/GFAP
6 m Regen.
Lesion
LGN
Mouse-1
Mouse-2
Mouse-3
Mouse-4
Mouse-5
Mouse-6
Mouse-7
B
Light OFF
Light ON
Light OFF
Light ON
M1:41.8%
M2:19.8%
M3:27%
M4:15.2%
M5:21.8%
M6:24.9%
M7:23%
PLR score
C
% of pupil constriction
@ 0.5 mW/cm² blue light
100
80
60
40
20
0
***
Saline
LPC
0 5 10 20 30 40 50 60
Time (s)
D
% of pupil constriction
@ 0.5 mW/cm² blue light
Saline
LPC
***
0 1 2 3 4 5
Time (s)
E
% of GFP+ RGCs
8
6
4
2
0
GFP+
F
MBP/GFP/Tuj1
Opn4-GFP mouse
G
GFP/MBP

Supplementary Fig. 11: Axon regeneration and PLR recovery in individual Pten/Socs3 knockout mice 6 months after pre-OPN OTI and optic tract demyelination impairs PLR.

(A) Representative images (left) showing CTB-labeled regenerated axons (green) 6 months after pre-OPN OTI in individual Pten/Socs3 knockout and CNTF expression mice. The lesion site was indicated by GFAP staining (red). Scale bar: 200μm.

Zoomed-in images (right) within the dashed rectangles showing CTB traced axons in the OPN regions. Scale bar: 50μm.

(B) Representative pupil images from individual regeneration mice before and after 1-minute light (0.5 mW/cm^2) stimulation. The PLR score indicates the percentage of pupil constriction. M1-M7 represents mouse1-mouse7 in (A).

(C) Quantification of the pupil constriction dynamics within 1 minute of light (0.5 mW/cm^2) stimulation after saline or LPC optic tract injection. Data presents mean ± SEM. n=5 mice. Statistical significance: $p<0.0001$ (5 s). ANOVA followed by Bonferroni test, two-sided.

(D) Quantification of pupil constriction dynamics within the first 5 sec in (C). Data presents mean ± SEM. n=5 mice. Statistical significance: p=0.0004, 0.0002, 0.0002 (1 s, 4 s, 5 s), $p<0.0001$ (2 s, 3 s). ANOVA followed by Bonferroni test, two-sided.

(E) Quantification of the percentage of GFP+ RGCs in Opn4-GFP mice.

(F-G) Cross sections (F) and longitude sections (G) of optic nerve from intact Opn4-GFP mice showing co-staining of GFP (green) and myelin marker MBP (red). The arrow indicates that the GFP labeled ipRGC axon was unmyelinated in the cross section (F). Tuj1 (blue) was used for staining the axons in nerves (F). Scale bar: 5 μm (F), 20 μm (G).

*** $p\leq0.001$

Source data are provided as a Source Data file.

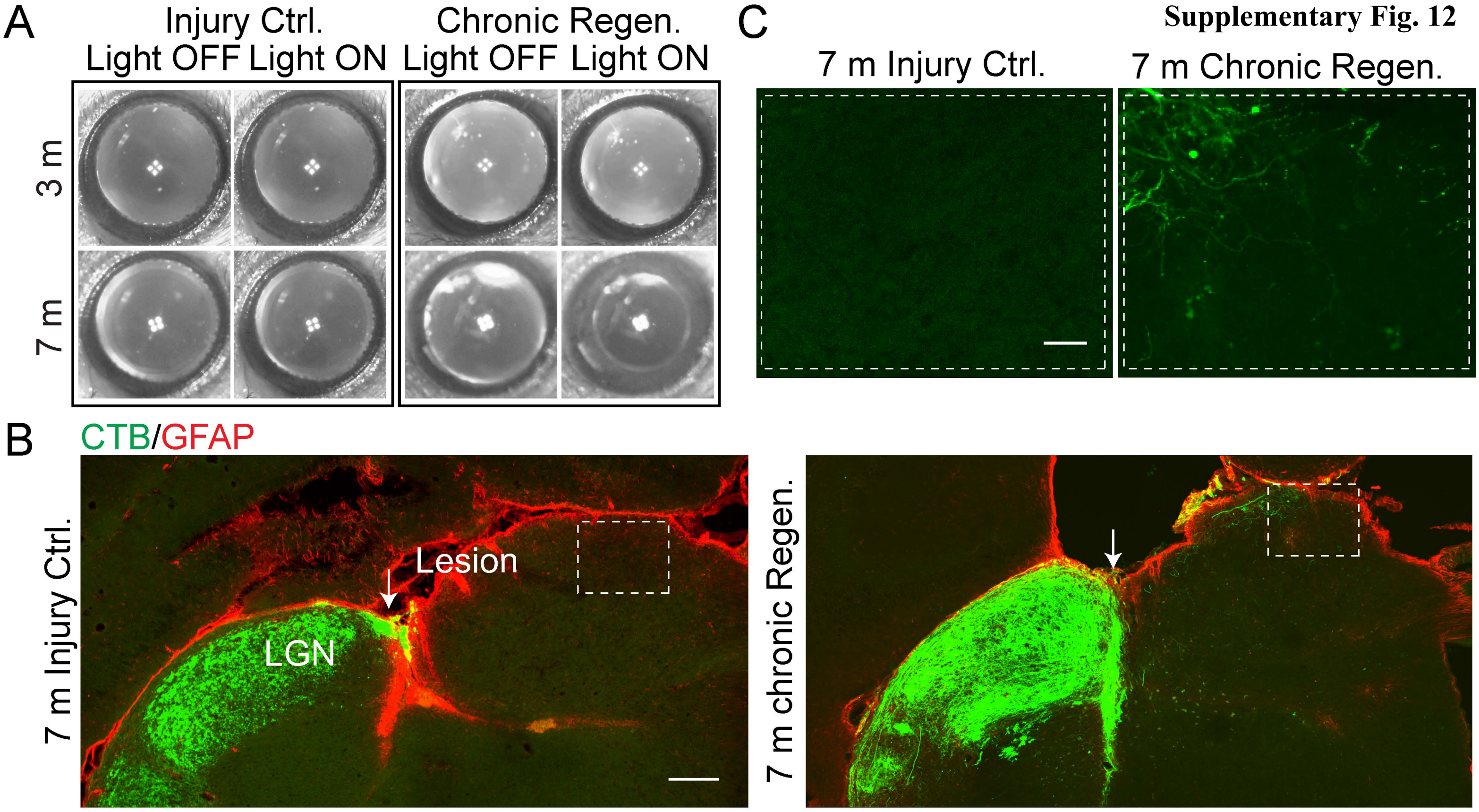
A
Injury Ctrl.
Chronic Regen.
Light OFF
Light ON
Light OFF
Light ON
3 m
7 m
B
CTB/GFAP
7 m Injury Ctrl.
Lesion
LGN
7 m chronic Regen.
C
7 m Injury Ctrl.
7 m Chronic Regen.

Supplementary Fig. 12: PSCL manipulation promotes axon regeneration and PLR recovery in chronic pre-OPN lesion model.

(A) Representative pupil images from the mice with chronic pre-OPN OTI before and after 1-minute light (0.5 mW/cm^2) stimulation. The chronic Regen. group displayed a partial PLR recovery 7 months post-injury.

(B) Representative images showing a lack of CTB-labeled RGC axon (green) regeneration in Injury Ctrl. mice undergoing a chronic pre-OPN OTI, while PSCL manipulation in Pten$^{f/f}$;Socs3$^{f/f}$ mice promotes axonal regrowth into OPN 7 months after chronic pre-OPN OTI. The lesion site was indicated by GFAP (red) staining. Scale bar: 200 μm.

(C) Zoomed-in images of the dashed rectangles in (B) display the OPN regions. Scale bar: 50 μm.

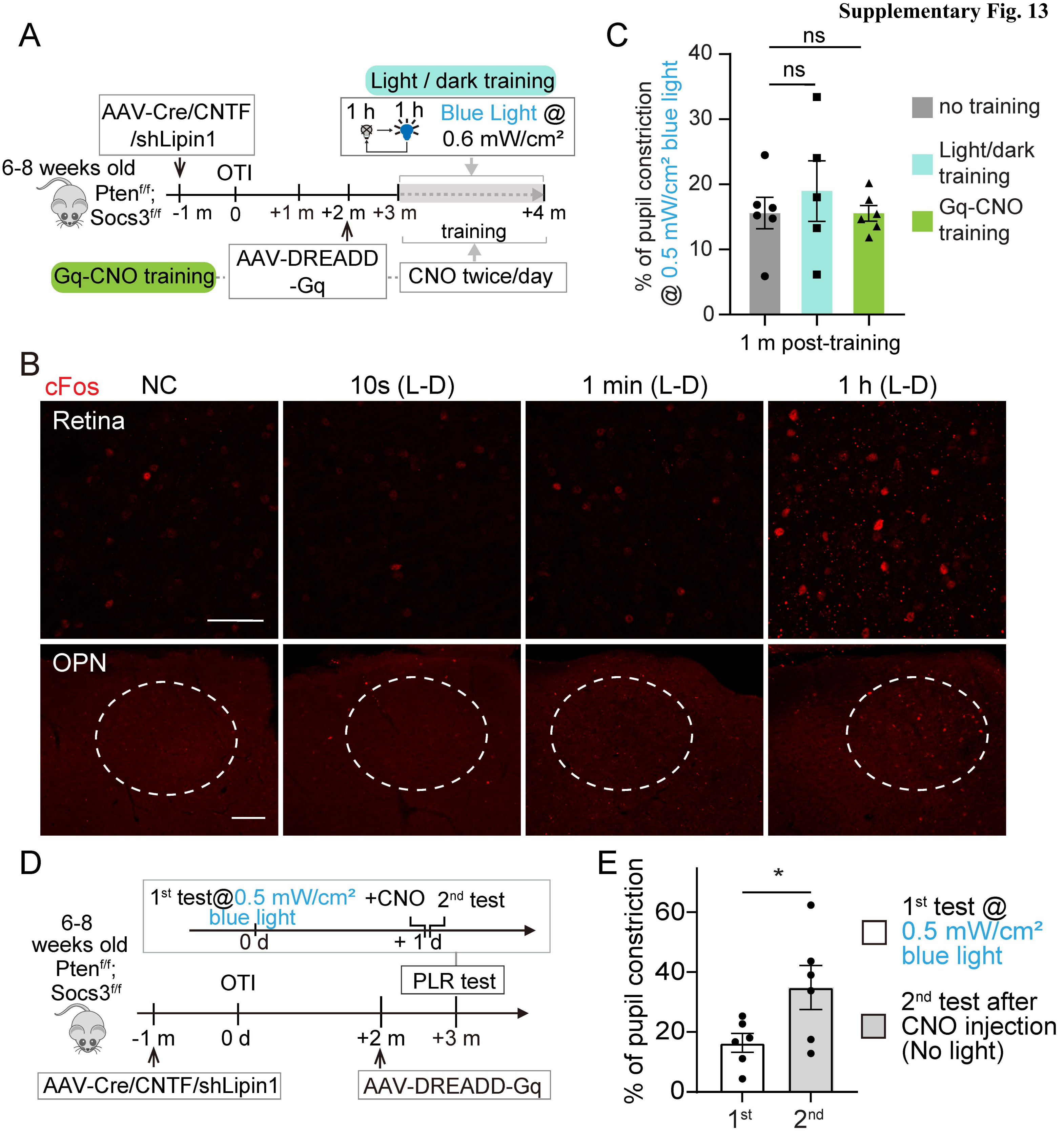
A
Light / dark training
1 h 1 h Blue Light @ 0.6 mW/cm²
AAV-Cre/CNTF /shLipin1
6-8 weeks old
Pten f/f; Socs3 f/f
OTI
-1 m 0 +1 m +2 m +3 m +4 m
training
Gq-CNO training
AAV-DREADD -Gq
CNO twice/day
C
ns
ns
% of pupil constriction @ 0.5 mW/cm² blue light
40 30 20 10 0
1 m post-training
no training
Light/dark training
Gq-CNO training
B
cFos
NC
10s (L-D)
1 min (L-D)
1 h (L-D)
Retina
OPN
D
1st test@0.5 mW/cm² blue light
+CNO
2nd test
0 d
+ 1 d
6-8 weeks old
Pten f/f; Socs3 f/f
OTI
PLR test
-1 m 0 d +2 m +3 m
AAV-Cre/CNTF/shLipin1
AAV-DREADD-Gq
E
*
% of pupil constriction
60 40 20 0
1st 2nd
1st test @ 0.5 mW/cm² blue light
2nd test after CNO injection (No light)

Supplementary Fig. 13: Light/dark training and DREADD-Gq training fail to improve the recovery of PLR.

(A) Schematic diagram showing the experimental design for 1-month neuronal modulation training starting from 3 months after injury in PSCL Regen. mice. The Light/dark training subjected mice to alternating cycles of 1-hour blue light (0.6 mW/cm^2) and 1 hour darkness. For Gq-CNO training, mice received AAV-DREADD-Gq intravitreal injection 2 months after injury, followed by bi-daily intraperitoneal injections of 5 mg/kg CNO starting at 3 months post-injury. PLR was assessed after completing the 1-month training.

(B) Representative images showing the cFos expression after different paradigms (Normal circadian (NC), 10 seconds, 1 minute, or 1 hour sustained light-dark cycle) of light stimulation in the retina (upper panel) and OPN (lower panel). Scale bar: 50 µm (upper panel), 100 µm (lower panel).

(C) Quantification of percentages of pupil constrictions following 1 minute of 0.5 mW/cm^2 light stimulation after training in different groups. Data presents mean ± SEM. n=5 (Light/dark training) and n=6 (no training and Gq-CNO training) mice per group. Statistical significance: p=0.6378 (no training vs. Light/dark training), p>0.999 (no training vs. Gq-CNO training). ANOVA followed by Dunnett's test, two-sided.

(D) Schematic diagram illustrating the experimental design for (E). PSCL Regen. mice received AAV-DREADD-Gq intravitreal injection 2 months post-injury. 1 month later, mice underwent the 1st PLR test with 0.5 mW/cm^2 blue light stimulation. 1 day later, in the absence of light stimulation, the 2nd PLR test was performed following one 5 mg/kg CNO intraperitoneal injection.

(E) Quantification of percentages of pupil constrictions during the 1st or 2nd PLR test. Data presents mean ± SEM. n=6 mice. Statistical significance: p=0.0339, student's t-test, two-sided.

ns, not significant p>0.05, * p≤0.05

Source data are provided as a Source Data file.

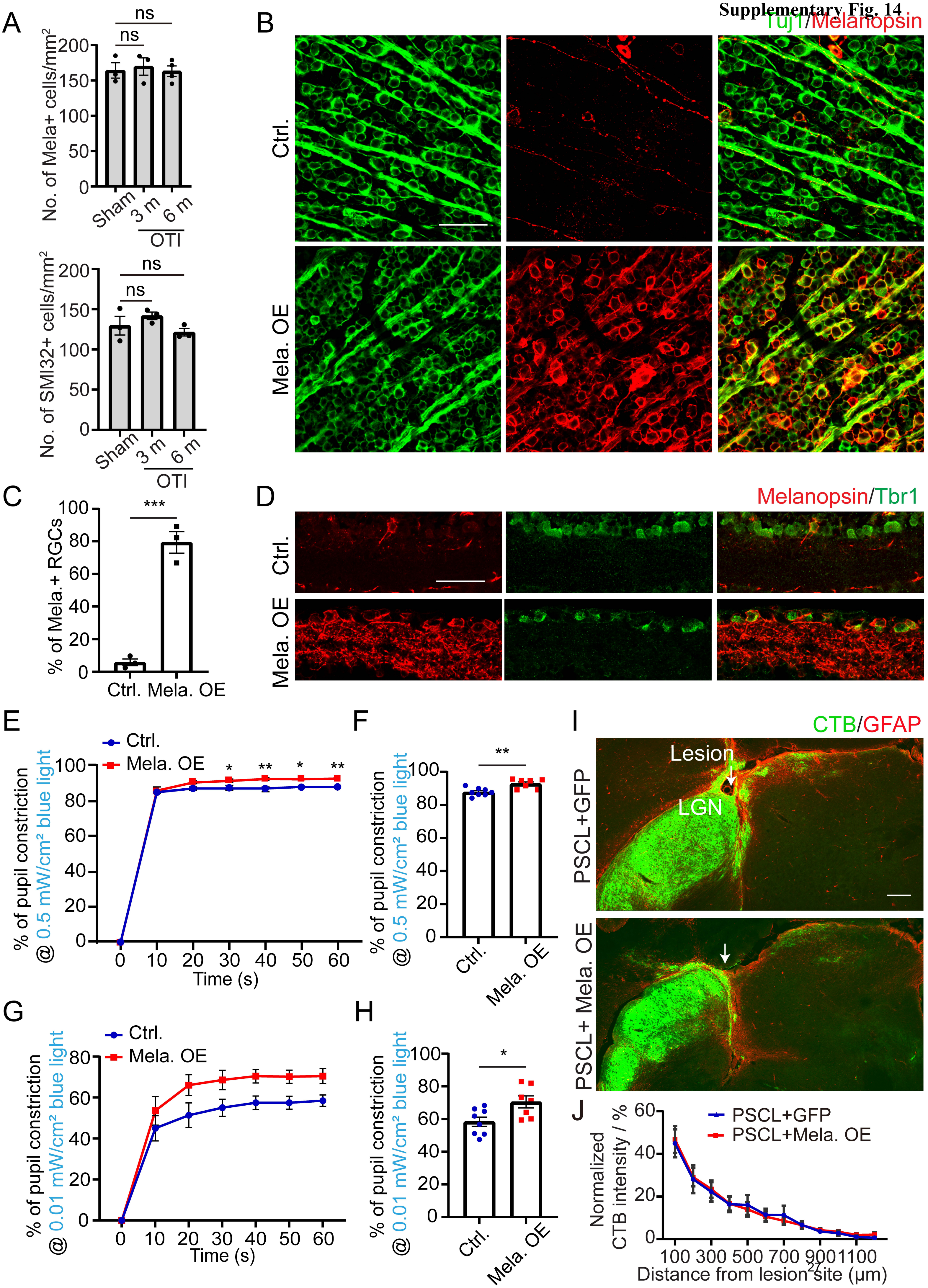
A
ns
ns
No. of Mela+ cells/mm²
200
150
100
50
0
Sham
3 m
6 m
OTI
No. of SMI32+ cells/mm²
B
Tuj1/Melanopsin
Ctrl.
Mela. OE
C
***
% of Mela.+ RGCs
100
80
60
40
20
Ctrl.
Mela. OE
D
Melanopsin/Tbr1
E
Ctrl.
Mela. OE
*
**
% of pupil constriction
@ 0.5 mW/cm² blue light
Time (s)
0
10
20
30
40
50
60
F
G
@ 0.01 mW/cm² blue light
H
I
CTB/GFAP
Lesion
LGN
PSCL+GFP
PSCL+ Mela. OE
J
Normalized
CTB intensity / %
PSCL+GFP
PSCL+Mela. OE
100 300 500 700 9001100
Distance from lesion site (μm)

Supplementary Fig. 14: Overexpression of melanopsin enhances PLR in intact mice and does not further promote axon regeneration induced by PSCL manipulation after OTI.

(A) Quantification of surviving ipRGCs labeled with Melanopsin (upper panel) and SMI32 (lower panel). Data presents mean ± SEM. For melanopsin labeling group: n=3 (Sham), 3 (3 m OTI), and 4 (6 m OTI) mice. Statistical significance: p=0.9003 (sham vs. 3 m OTI), 0.9949 (sham vs. 6 m OTI). For SMI32 labeling group: n=3 each group. Statistical significance: p=0.4755 (sham vs. 3 m OTI), 0.6965 (sham vs. 6 m OTI), ANOVA followed by Dunnett test, two-sided.

(B) Flat mount retina images showing the Tuj1 (green) and melanopsin (red) staining in Ctrl. and Mela. OE group. Scale bar: 50 µm.

(C) Quantification of the percentage of melanopsin+ RGCs in Ctrl. and Mela. OE groups. Data presents mean± SEM. n=3 mice. Statistical significance: p=0.0004, student's t-test, two-sided.

(D) Representative images showing the melanopsin (red) and Tbr1 (green) staining. Scale bar: 50 µm.

(E-H) Quantification of pupil constriction dynamics within 1 minute of light stimulation (E, G) and pupil constriction after 1 minute of light stimulation (F, H) in Ctrl. and Mela. OE groups at light intensities of 0.5 mW/cm^2 (E, F) and 0.01 mW/cm^2 (G, H). Data presents mean ± SEM. n=8 (Ctrl.), n=7 (Mela. OE). Statistical analysis: p=0.0405, 0.0015, 0.0119, 0.0069 (E 30-60 s). No significant differences between the Ctrl. and Mela. OE groups at any time point in (G). ANOVA followed by Bonferroni test in (E) and (G); Statistical significance: p=0.0026 (F), p=0.0204 (H), student's t-test in (F) and (H), two-sided.

(I) Representative images of CTB-traced axons (green) 3 months after pre-OPN OTI with lesion sites indicated by GFAP (red) staining. Scale bar: 200 µm.

(J) Quantification of the percentage of normalized CTB intensity at different distances from the lesion site. No significant difference exists between the two groups. Data presents mean ± SEM. n=12 mice, ANOVA followed by Bonferroni test, two-sided.

ns, not significant $p>0.05$, * $p\leq0.05$, ** $p\leq0.01$, *** $p\leq0.001$

Source data are provided as a Source Data file.

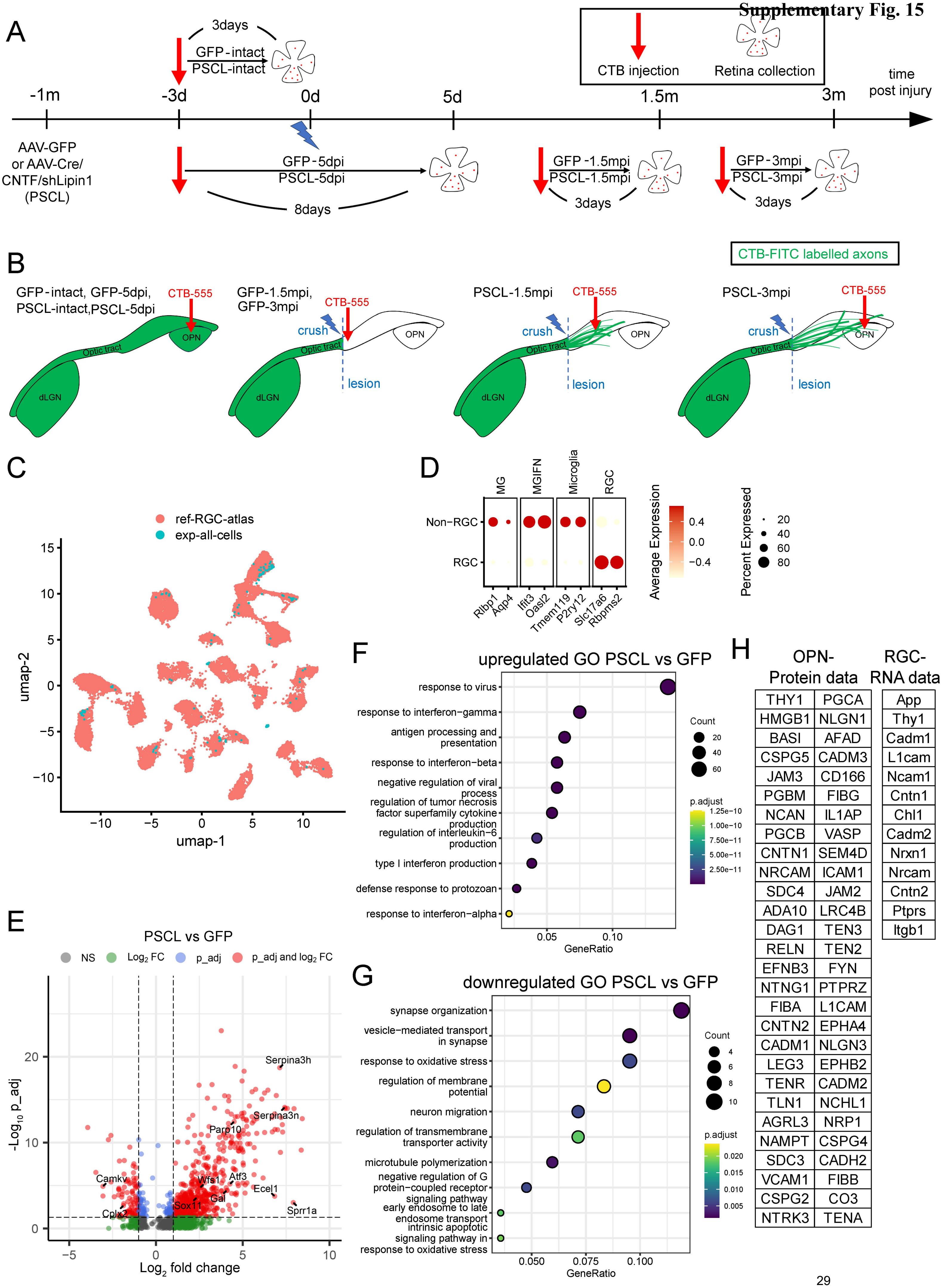
A
3days
GFP-intact
PSCL-intact
CTB injection
Retina collection
time post injury
-1m
-3d
0d
5d
1.5m
3m
AAV-GFP or AAV-Cre/ CNTF/shLipin1 (PSCL)
GFP-5dpi
PSCL-5dpi
8days
GFP-1.5mpi
PSCL-1.5mpi
3days
GFP-3mpi
PSCL-3mpi
3days
B
CTB-FITC labelled axons
GFP-intact, GFP-5dpi, PSCL-intact,PSCL-5dpi
CTB-555
OPN
Optic tract
dLGN
GFP-1.5mpi, GFP-3mpi
crush
lesion
PSCL-1.5mpi
PSCL-3mpi
C
ref-RGC-atlas
exp-all-cells
umap-2
umap-1
D
MG
MGIFN
Microglia
RGC
Non-RGC
RGC
Rlbp1
Aqp4
Ifit3
Oasl2
Tmem119
P2ry12
Slc17a6
Rbpms2
Average Expression
Percent Expressed
E
PSCL vs GFP
NS
Log2 FC
p_adj
p_adj and log2 FC
Serpina3h
Serpina3n
Parp10
Camkv
Wfs1
Atf3
Ecel1
Gal
Sox11
Cplx2
Sprr1a
-Log10 p_adj
Log2 fold change
F
upregulated GO PSCL vs GFP
response to virus
response to interferon−gamma
antigen processing and presentation
response to interferon−beta
negative regulation of viral process
regulation of tumor necrosis factor superfamily cytokine production
regulation of interleukin−6 production
type I interferon production
defense response to protozoan
response to interferon−alpha
Count
p.adjust
GeneRatio
G
downregulated GO PSCL vs GFP
synapse organization
vesicle−mediated transport in synapse
response to oxidative stress
regulation of membrane potential
neuron migration
regulation of transmembrane transporter activity
microtubule polymerization
negative regulation of G protein−coupled receptor signaling pathway
early endosome to late endosome transport
intrinsic apoptotic signaling pathway in response to oxidative stress
Count
p.adjust
GeneRatio
H
OPN- Protein data
THY1 PGCA
HMGB1 NLGN1
BASI AFAD
CSPG5 CADM3
JAM3 CD166
PGBM FIBG
NCAN IL1AP
PGCB VASP
CNTN1 SEM4D
NRCAM ICAM1
SDC4 JAM2
ADA10 LRC4B
DAG1 TEN3
RELN TEN2
EFNB3 FYN
NTNG1 PTPRZ
FIBA L1CAM
CNTN2 EPHA4
CADM1 NLGN3
LEG3 EPHB2
TENR CADM2
TLN1 NCHL1
AGRL3 NRP1
NAMPT CSPG4
SDC3 CADH2
VCAM1 FIBB
CSPG2 CO3
NTRK3 TENA
RGC- RNA data
App
Thy1
Cadm1
L1cam
Ncam1
Cntn1
Chl1
Cadm2
Nrxn1
Nrcam
Cntn2
Ptprs
Itgb1

Supplementary Fig. 15: Sequential labeling and marker expression analysis in single-cell RNA sequencing and axon guidance-related molecules identified in proteomics.

(A, B) Illustration of timeline for experimental design in single-cell RNA-seq, encompassing the tracing process, injury, and RGC collection. CTB-555 was injected into different positions to label targeted RGCs.

(C) Umap plot showing the integration result of cells collected in this study and a referenced RGC atlas [53] using the FIRM algorithm [96].

(D) Dot plot showing the expression of selected cell-type marker expression of filtered RGCs and non-RGCs. Noticed that filtered RGCs showed high expression for RGC marker genes while non-RGCs showed high expression for glia marker genes.

(E) Volcano plot displaying the differentially expressed genes between the PSCL group and the Injury Ctrl. group. Genes denoted by red dots exhibit a significant change in expression, as determined by a log2 fold change > 1 or < -1, and an adjusted p-value < 0.05.

(F, G) Dot plots illustrating the enriched Gene Ontology (GO) terms associated with the upregulated (panel F) and downregulated (panel G) genes, respectively, in the PSCL vs. GFP comparison, highlighting the biological processes that are differentially regulated between these two groups.

(H) Listing the potential ligand-receptor pairs mediating the reinnervation of OPN by regenerated axons based on the paired expression of RNA in RGCs and protein in OPN region.
Source data are provided as a Source Data file.
