# Supplementary material for: Functional optic tract rewiring via subtype- and target-specific axonal regeneration and presynaptic activity enhancement
Source: Nat Commun. 2025 Mar 4;16:2174. doi: 10.1038/s41467-025-57445-x (PMC11880380; doi:10.1038/s41467-025-57445-x)
Supplement: Supplementary file 2 — Description of Additional Supplementary Files [file 41467_2025_57445_MOESM2_ESM.docx]

**Description of Additional Supplementary Files**

**Supplementary Movie 1 (related to Fig. 1): Scheme of the OTI model.**

The video illustrates the surgical procedures for performing the OTI model on one side of mouse brain. The mouse brain, forceps tips and injury tract were created by modifying the code from “Neuropixels trajectory explorer” program by Andy Peters.

**Supplementary Movie 2 (related to Fig. 1): Example recording of PLR responses observed in Sham and one-day after pre-OPN OTI mice.**

The mice were subjected to a 1-minute stimulation under a blue light (0.5 mW/cm^2^). The left panel displays a normal PLR response in the Sham mouse, while the right panel shows no response in the pre-OPN OTI mouse. Time is presented as hr:min:sec.

**Supplementary Movie 3 (related to Fig.3): Example recording of PLR responses observed in 6 months injury control mouse and 6 months regeneration mouse.**

The control mouse (left) demonstrated no significant pupil constriction in response to 1-minute light (0.5 mW/cm^2^) stimulation. In contrast, the regeneration mouse with Pten/Socs3 knockout and CNTF expression (right) showed partial recovery of PLR 6 months after the pre-OPN OTI. Time is presented as hr:min:sec.

**Supplementary Movie 4 (related to Fig. 6): Example recording of PLR responses observed in 3 months PSCL regeneration mice before and after i.p. injection of 5 mg/kg R-roscovitine.**

The PSCL regeneration mouse displayed partial PLR recovery under a 1-minute light (0.5 mW/cm^2^) stimulation 3 months after the pre-OPN OTI (left). The application of R-roscovitine further increased the PLR response in the regenerated mouse (right). Time is presented as hr:min:sec.
