# Supplementary material for: Functional optic tract rewiring via subtype- and target-specific axonal regeneration and presynaptic activity enhancement
Source: Nat Commun. 2025 Mar 4;16:2174. doi: 10.1038/s41467-025-57445-x (PMC11880380; doi:10.1038/s41467-025-57445-x)
Supplement: Supplementary file 7 — Reporting Summary [file 41467_2025_57445_MOESM7_ESM.pdf]

nature portfolio

Corresponding author(s): Kai LIU

Last updated by author(s): Jan 31, 2025

Reporting Summary

Nature Portfolio wishes to improve the reproducibility of the work that we publish. This form provides structure for consistency and transparency in reporting. For further information on Nature Portfolio policies, see our Editorial Policies and the Editorial Policy Checklist.

Statistics

For all statistical analyses, confirm that the following items are present in the figure legend, table legend, main text, or Methods section.

n/a	Confirmed	
[ ]	[x]	The exact sample size (*n*) for each experimental group/condition, given as a discrete number and unit of measurement
[ ]	[x]	A statement on whether measurements were taken from distinct samples or whether the same sample was measured repeatedly
[ ]	[x]	The statistical test(s) used AND whether they are one- or two-sided<br>*Only common tests should be described solely by name; describe more complex techniques in the Methods section.*
[ ]	[x]	A description of all covariates tested
[ ]	[x]	A description of any assumptions or corrections, such as tests of normality and adjustment for multiple comparisons
[ ]	[x]	A full description of the statistical parameters including central tendency (e.g. means) or other basic estimates (e.g. regression coefficient) AND variation (e.g. standard deviation) or associated estimates of uncertainty (e.g. confidence intervals)
[ ]	[x]	For null hypothesis testing, the test statistic (e.g. *F*, *t*, *r*) with confidence intervals, effect sizes, degrees of freedom and *P* value noted<br>*Give P values as exact values whenever suitable.*
[x]	[ ]	For Bayesian analysis, information on the choice of priors and Markov chain Monte Carlo settings
[x]	[ ]	For hierarchical and complex designs, identification of the appropriate level for tests and full reporting of outcomes
[x]	[ ]	Estimates of effect sizes (e.g. Cohen's *d*, Pearson's *r*), indicating how they were calculated

Our web collection on statistics for biologists contains articles on many of the points above.

Software and code

Policy information about availability of computer code

Data collection	Fluorescent images were collected using Confocal microscopy Zeiss, LSM 880. EM images were collected using Hitachi H-7650 transmission electron microscope. The pupil images were obtained by an infrared camera. Light intensity was determined by THORLABS, PM100D. In vivo electrophysiology recording was conducted by a Plexon system (Plexon Inc., Dallas, Texas). RNA-seq data was obtained using the NovaSeq PE150 platform. Proteomics data was obtained using a Bruker timsTOF Pro mass spectrometer.
Data analysis	Images of the immunohistochemistry samples were processed and analyzed using ImageJ software.<br>Statistical analyses were performed using GraphPad Prism 9 software.<br>RNA-Seq data were processed and analyzed with STAR, RSeQC, RStudio, Metascape, Cytoscape, and GSEA software. Raw timsTOF PASEF data were processed through FragPipe for peptide identification, FDR filtering, and label-free quantification. Proteomics data was analyzed with RStudio software and "MSstats" package.

For manuscripts utilizing custom algorithms or software that are central to the research but not yet described in published literature, software must be made available to editors and reviewers. We strongly encourage code deposition in a community repository (e.g. GitHub). See the Nature Portfolio guidelines for submitting code & software for further information.

Data

Policy information about availability of data

All manuscripts must include a data availability statement. This statement should provide the following information, where applicable:

- Accession codes, unique identifiers, or web links for publicly available datasets
- A description of any restrictions on data availability
- For clinical datasets or third party data, please ensure that the statement adheres to our policy

The RNA-seq data generated in this study are publicly available through GEO repositories (NCBI GEO: GSE267938). The mass spectrometry proteomics data have been deposited to the ProteomeXchange Consortium (https://proteomecentral.proteomexchange.org) via the iProX partner repository 107,108 with the dataset identifier PXD052573. The RNA-seq data utilized but not generated in this study are available at GEO repositories (NCBI GEO: GSE137400). Any additional information required to reanalyze the data reported in this paper is available from the lead contact upon reasonable request. Source Data are provided with this paper.

Research involving human participants, their data, or biological material

Policy information about studies with human participants or human data. See also policy information about sex, gender (identity/presentation), and sexual orientation and race, ethnicity and racism.

Reporting on sex and gender	No human participants were involved in this study.
Reporting on race, ethnicity, or other socially relevant groupings	No human participants were involved in this study.
Population characteristics	No human participants were involved in this study.
Recruitment	No human participants were involved in this study.
Ethics oversight	No human participants were involved in this study.

Note that full information on the approval of the study protocol must also be provided in the manuscript.

Field-specific reporting

Please select the one below that is the best fit for your research. If you are not sure, read the appropriate sections before making your selection.

[x] Life sciences   [ ] Behavioural & social sciences   [ ] Ecological, evolutionary & environmental sciences

For a reference copy of the document with all sections, see nature.com/documents/nr-reporting-summary-flat.pdf

Life sciences study design

All studies must disclose on these points even when the disclosure is negative.

Sample size	The sample sizes used in this study were not predetermined using statistical methods. Instead, they were estimated based on previous experience and other publications. The sample sizes were indicated in the legend of each Figure and Extended Figure.
Data exclusions	Mice with LGN damage from the OTI surgery were excluded from further quantification and analysis.<br>Mice with pupils respond to light stimulation after pre-OPN OTI were excluded from further analysis.<br>In PRV tracing, if any mouse from either group showed a large area of GFP-positive signals in the margin of the retina of the PRV-injected eye, this would indicate that the PRV had diffused into the vitreous, and the mouse would be excluded from the analysis.<br>RNA-Seq samples with low cDNA quality or low expression of both Sncg and Rbpms genes were excluded from downstream library construction.
Replication	The experimental findings in this study were reliably reproduced. All experiments were repeated multiple times with independent mice, with most of them replicated more than three times. The number of animals used for statistical analysis is described in the corresponding figure legends. All attempts at replication were successful.
Randomization	The mice in all experiments were randomly assigned to receive indicated treatments, as described in the methods and figure legends.
Blinding	During the experiments, all surgeries and behavior analyses were carried out in a blinded manner to the treatment of the mice. For image analysis, the quantification was done in a blinded manner as well.

Reporting for specific materials, systems and methods

We require information from authors about some types of materials, experimental systems and methods used in many studies. Here, indicate whether each material, system or method listed is relevant to your study. If you are not sure if a list item applies to your research, read the appropriate section before selecting a response.

Materials & experimental systems

n/a	Involved in the study
[ ]	[x] Antibodies
[x]	[ ] Eukaryotic cell lines
[x]	[ ] Palaeontology and archaeology
[ ]	[x] Animals and other organisms
[x]	[ ] Clinical data
[x]	[ ] Dual use research of concern
[x]	[ ] Plants

Methods

n/a	Involved in the study
[x]	[ ] ChIP-seq
[ ]	[x] Flow cytometry
[x]	[ ] MRI-based neuroimaging

Antibodies

Antibodies used	Primary antibodies: Rabbit anti-FITC (Invitrogen, 71-1900), Mouse or Rabbit anti-Tuj1 (BioLegend, 801202 and 802001), Mouse anti-SMI32 (BioLegend, 801701), Rabbit anti-Melanopsin (Advanced Targeting System, AB-N39), Mouse anti-GFAP (BioLegend, 808402), Rabbit anti-c-Fos (Cell Signaling Technology, 2250), Chicken anti-GFP (Invitrogen, A10262), Rabbit anti-Homer1 (Millipore, ABN37), Mouse anti-Bassoon (Abcam, ab82958), Chicken-anti-Tbr1 (Millipore, AB2261), Chicken anti-Tbr2 (Millipore, AB15894), Mouse anti-MBP (BioLegend, 808402), Rabbit anti-Phospho-c-Jun (Cell Signaling Technology, 3270), Mouse anti-Phospho-c-Jun (Invitrogen, MA5-27760), Mouse anti-parvalbumin (Millipore Sigma, P3088).<br>Secondary antibodies: Goat anti-Mouse 555 (Invitrogen, A-21424), Goat anti-Mouse 488 (Invitrogen, A-11029), Goat anti-Mouse Cy5 (Invitrogen, A-10524), Goat anti-Rabbit 555 (Invitrogen, A-21429), Goat anti-Rabbit 488 (Invitrogen, A-11034), Goat anti-Rabbit Cy5 (Invitrogen, A-10523), Goat anti-Chicken 647 (Invitrogen, A-21449) Goat anti-biotin (Thermo Fisher Scientific, B-2770).
Validation	To ensure the specificity of the antibodies used in this study, they were obtained from commercial suppliers that validated their specificity for the intended application, which included immunohistochemistry and western blotting. The validation information for the antibodies is reported on the suppliers' websites.

Animals and other research organisms

Policy information about studies involving animals; ARRIVE guidelines recommended for reporting animal research, and Sex and Gender in Research

Laboratory animals	C57BL/6J mice were obtained from Charles River. The Ptenf/f; Socs3f/f double-floxed mice were provided as a gift by Prof. Zhigang He (Boston Children's Hospital). Opn4Cre mice were obtained from Prof. Samer Hattar at Johns Hopkins University. The Opn4Cre mice were crossed with Ptenf/f;Socs3f/f mice to generate Opn4Cre;Ptenf/f;Socs3f/f mice. Opn4-GFP mice were obtained from the Mutant Mouse Regional Resource Center, an NIH funded strain repository. Vglut2-Cre mice were purchased from Jackson Laboratory. 6-8 weeks old mice of both genders were used in this study.
Wild animals	No wild animals were involved in this study.
Reporting on sex	Both male and female animals were used in this study. The sex of the animals was not considered in the study design because, based on our experience, it does not affect axon regeneration and functional restoration after CNS injury. Therefore, sex-based analyses were not conducted.
Field-collected samples	This study did not involve samples collected from the field.
Ethics oversight	All experiments were conducted in compliance with the guidelines of the Laboratory Animal Facility at the Hong Kong University of Science and Technology.

Note that full information on the approval of the study protocol must also be provided in the manuscript.

Plants

Seed stocks	No plants were involved in this study.
Novel plant genotypes	No plants were involved in this study.
Authentication	No plants were involved in this study.

Flow Cytometry

Plots

Confirm that:

- [ ] The axis labels state the marker and fluorochrome used (e.g. CD4-FITC).
- [ ] The axis scales are clearly visible. Include numbers along axes only for bottom left plot of group (a 'group' is an analysis of identical markers).
- [ ] All plots are contour plots with outliers or pseudocolor plots.
- [ ] A numerical value for number of cells or percentage (with statistics) is provided.

Methodology

Sample preparation	AAV-DIO-GFP was injected into the vitreous body of vGlut2-cre mice eye to label RGCs one month before any injury was applied to the animals. The RGC-labeled retina was quickly dissected out from mice after cervical dislocation and incubated with 0.5 mg/ml papain (Sigma-Aldrich, P4762) at 37 °C for 30 minutes. The retina was then washed with Neurobasal-A medium and triturated into a single-cell suspension by pipetting. After centrifugation at 300 g for 6 minutes at 4 °C, the retinal cells were resuspended in Neurobasal-A medium supplemented with 10% B27. For bulk RNAseq, fluorescence-activated cell sorting (FACS) was used to isolate labeled RGCs.
Instrument	BD FACS Aria III or IIIu Cell Sorter & Analyzer
Software	BD FACSDiva software
Cell population abundance	Based on previous experience, using qPCR or imaging to check for GFP mRNA expression or GFP fluorescence, GFP+ cells account for at least 80% of the post-sort fractions.
Gating strategy	FACS gating was performed to isolate the desired population by excluding small FSC dots and drawing a triangle area to include most cell populations with large FSC and small SSC, then excluding dead cells (DAPI-negative), selecting singlet cells, and then gating on GFP-positive cells to enrich the GFP-expressing population. The boundary for 'GFP Positive' and 'Negative' was determined by using GFP-negative and GFP-positive samples.

- [ ] Tick this box to confirm that a figure exemplifying the gating strategy is provided in the Supplementary Information.
